# Developing mRNA lipid nanoparticle vaccine effective for cryptococcosis in a murine model

**DOI:** 10.1038/s41541-025-01079-z

**Published:** 2025-02-04

**Authors:** Yeqi Li, Suresh Ambati, Richard B. Meagher, Xiaorong Lin

**Affiliations:** 1 Department of Microbiology, University of Georgia, Athens, GA 30602 USA; 2 Department of Genetics, University of Georgia, Athens, GA 30602 USA

**Keywords:** RNA vaccines, Fungal infection, Vaccines

## Abstract

*Cryptococcus neoformans* is considered a critical fungal pathogen by the World Health Organization and developing a vaccine for cryptococcosis is a top priority. Here, we employed mRNAs encoding an antigen(s) packaged in lipid nanoparticles (LNPs) to develop vaccines for cryptococcosis. Remarkably, when coupled with capsule adjuvant, vaccination with *CDA1*-LNPs protected the vast majority of mice from the otherwise lethal cryptococcosis. These results support the promise of mRNA-LNP vaccines against fungal diseases.

The environmental fungus *Cryptococcus neoformans* causes fatal meningoencephalitis in immunocompromised individuals, which claims over 112,000 lives annually^1,2^. Even with antifungal treatment, the global mortality rate of this disease is about 70%^1^. About 90% of relapse cases are caused by the original isolates^3^, pointing to failure of eradication by the antifungal therapy alone. The estimative cost of hospital in-patient care is $40,000–$70,000 for one patient in the U.S.^4,5^, highlighting the burden of cryptococcosis on our medical system. The World Health Organization (WHO) recently listed *C. neoformans* as one of the four critical fungal pathogens^6^, where research for treatment and prevention is urgently needed. Vaccination is one of the most effective ways to control infectious diseases^7^. Research on the development of cryptococcal vaccines has steadily progressed over the years, including whole cell vaccines and subunit recombinant protein vaccines using cryptococcal antigens such as *CDA1* (chitin deacetylase 1) and *BLP4* (barwin-like domain protein 4), as elegantly discussed recently^7,8^. However, no cryptococcal vaccine has emerged successfully yet for clinical testing. Due to major scientific and technological breakthroughs and the establishment of mRNA-vaccine infrastructures, stabilized mRNAs with modified uridine (e.g. N1-methylpseudouriding or m1ψ) encoding immunogens packaged in and delivered through LNPs have become a clinically proven and effective vaccine technology^9–11^. Here we constructed mRNA-LNPs that encode known cryptococcal antigens. We aimed to provide the proof-of-principal evidence that mRNA-LNP vaccines can protect against a highly complex and deadly fungal pathogen in a murine model of cryptococcosis.

## Assembly and validation of mRNA-LNPs

**Fig. 1:**
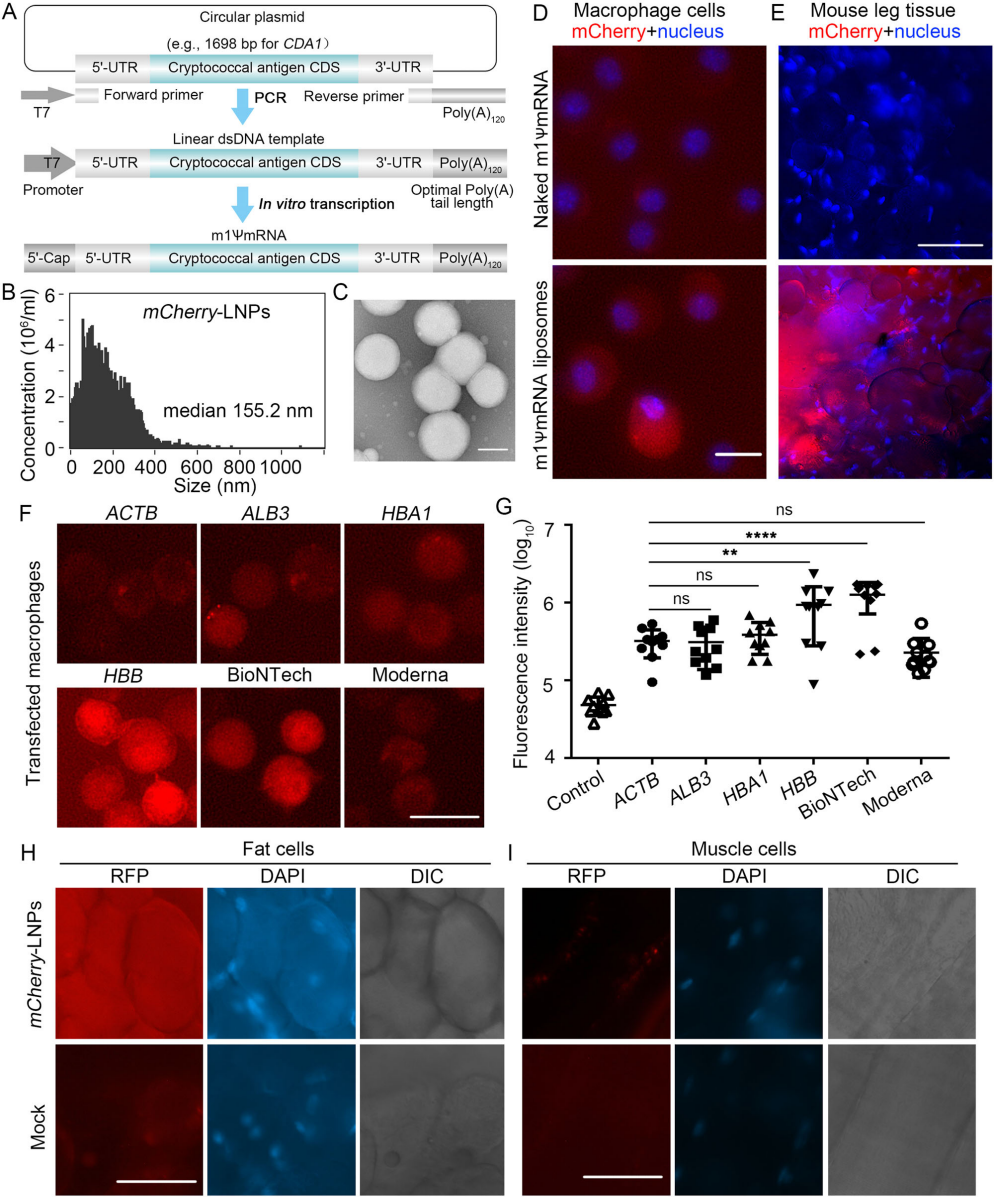
Validation of mRNA-LNPs using the mCherry reporter. **A** The process of making mRNAs encoding cryptococcal immunogens: synthesis of codon optimized cryptococcal antigen genes, PCR amplification of linear DNA templates for transcription, and in vitro transcription of the m1ψ base modified polyadenylated mRNAs. **B** *mCherry*-LNP size distribution was analyzed by NanoFCM Flow Nanoanalyzer. **C** *mCherry*-LNP size distribution was analyzed by electron microscopy. **D** 2.5 μg naked *mCherry* mRNA or *mCherry*-LNPs were transfected in murine macrophage J774A.1 cells and incubated for 24 h. Cells were stained with 1 μg/ml Hoechst 33342 for 20 min. Scale bar: 20 μm. **E** At 24 h post intramascular injection with 2 μg mCherry-LNPs into the thighs of mice, muscle with associated subcutaneous fat tissue surrounding the injection sites were excised. Cells were stained with 1 μg/ml Hoechst 33342 for 20 min. Scale bar: 90 μm. The images (**F**) and quantification (**G**) of the mCherry signal in macrophage cells at 24 h after transfection with 2 μg *mCherry*-LNPs with different 5’ UTRs. Scale bar: 20 μm. After i.m. injection with 2 μg mCherry-LNPs using BioNTech 5’ UTR into the thighs of mice for 24 h, subcutaneous fat tissues (**H**) and muscle (**I**) surrounding the injection sites were excised. Cells were stained with 1 μg/ml Hoechst 33342 for 20 min. Scale bar: 90 μm. Statistical significance was determined using a one-way ANOVA statistical analysis. ns, not significant; **p < 0.01; ****p < 0.0001.

We employed the following path in making our mRNAs (Fig. 1A). During the microfluidics preparation of mRNA-LNPs, we started with rapid mixing of mRNA solution and a lipid mixture containing an ionizable lipid, a helper lipid, cholesterol, and a PEG lipid^12^. Immediately after the assembly, they were dialyzed into a neutral buffer to convert mRNA lipid complexes into stable mRNA-LNPs^12^. We tested packaging small yeast tRNAs or yeast total RNAs and observed 80–95% encapsulation efficiency (Supplementary Fig. 1A).

Following this protocol, we prepared *mCherry*-LNPs and obtained 84–97% packaging efficiencies (Supplementary Fig. 1B). The median diameter of the *mCherry*-LNPs was ~155 nm (Fig. 1B, C, Supplementary Fig. 1C). To examine its biological activity, we incubated naked *mCherry* RNA or *mCherry*-LNPs with macrophage cells. In the naked mRNA transfected cells, we did not observe any significant fluorescence signal compared to the non-transfected control cells (Fig. 1D and Supplementary Fig. 1D). By contrast, more than 90% of the macrophage cells showed red fluorescence with *mCherry*-LNPs transfection. We then injected these mRNA-LNPs intramuscularly into mice and dissected thigh tissue at the injection site after 24 h. Thigh tissue from mock injected mice were used as a control. As expected, the control tissue showed no significant red fluorescence (Fig. 1E). By contrast, approximately 10% of the fields from *mCherry*-LNPs injected mice showed clusters of strongly red fluorescent cells with large round morphologies consistent with being adipocytes (Fig. 1E and Supplementary Fig. 1E). These results confirmed that mRNA-LNPs were taken up, and that mRNAs were released and translated by host cells ex vivo and in vivo.

## Optimization of the 5’ UTR enhances protein production

Since using minimal amount of mRNA reduces vaccine cost, we tested *mCherry* constructs with six 5’ UTRs: *ACTB*, *ALB3*, *HBA1*, *HBB* and those from BioNTech and Moderna COVID vaccines^9^. We found that *mCherry*-LNPs using the 5’ UTR from *ALB3*, *HBA1* and Moderna showed a similar level of fluorescence as our original *ACTB* 5’ UTR in macrophage cells (Fig. 1F, G). Remarkably, *mCherry*-LNPs using the 5’ UTR from *HBB* and BioNTech showed a 4-fold increase in the mCherry intensity. This result highlights the known role of 5’ UTR in mRNA expression^13^. We decided to use 5’ UTR from the BioNTech for the following experiments due to its ability to drive higher and more uniform expression (Supplementary Fig. 1F). To verify that *mCherry*-LNPs using the 5’ UTR from BioNTech would also improve protein expression in vivo, we injected *mCherry*-LNPs into mice and checked the dissected tissues for red fluorescence. The adipocytes adjacent to the injected tissues showed higher and more uniform mCherry expression (Fig. 1H and Supplementary Fig. 1G). Moreover, we found successful mCherry expression in adjacent muscle cells, which was not observed with the original *ACTB* 5’ UTR construct (Fig. 1I and Supplementary Fig. 1G).

## *CDA1* mRNA-LNPs with capsule offers strong protection against cryptococcosis

Antigen selection is particularly important as fungi express thousands of secreted proteins that could be immunogenic. Here we selected Cda1 and Blp4 to construct mRNA-LNPs: (i) *CDA1* and *BLP4* transcripts ranked the 32^nd^ and 264^th^ among several thousand detected cryptococcal gene transcripts in mouse lungs based on our analyses of the published RNA-seq data^14^ (Supplementary Fig. 2A, B). (ii) Cda1 and Blp4 subunit protein vaccines delivered in glucan particles offered good protection in mouse models^8^.

**Fig. 2:**
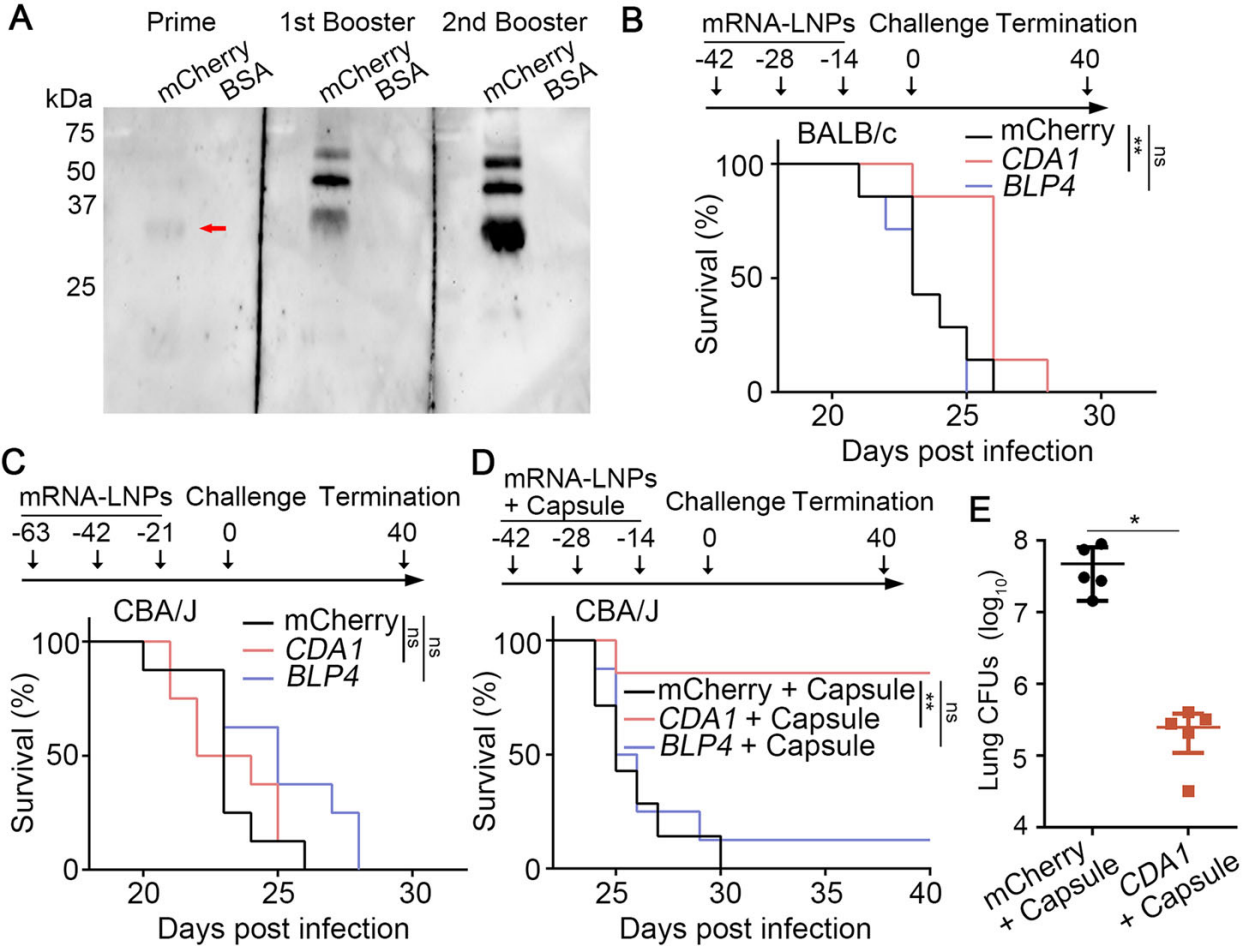
Vaccination with *CDA1*-LNPs+capsule protects mice from subsequent lethal challenge of cryptococcosis. **A** The antibody production was analyzed by western blot with purified mCherry protein (monomer: 26 kDa). **B** Survival rates of BALB/c mice vaccinated with *mCherry*-LNPs, *CDA1*-LNPs, *BLP4*-LNPs and then challenged with H99 intranasally. Eight mice were included in each group. **C** Survival rates of CBA/J mice vaccinated with *mCherry*-LNPs, *CDA1*-LNPs, *BLP4*-LNPs and then challenged with H99. Eight mice were included in each group. **D** Survival rates of CBA/J mice vaccinated with *mCherry*-LNPs+ capsule (250 μg), *CDA1*-LNPs+capsule (250 μg), *BLP4*-LNPs+capsule (250 μg), and then challenged with H99. Seven mice were included in each group. Survival curves are from two independent experiments. **E** The fungal burdens in lungs of moribund control mice at the time of euthanasia and mice vaccinated *CDA1*-LNPs+capsule at time of termination (DPI 40). The statistical significance of animal survival between groups was determined by the Mantel-Cox log-rank test. ns, not significant; **, *p* < 0.001.

Thus, we prepared *BLP4*-LNPs and *CDA1*-LNPs following the same protocol with 84–97% packaging efficiencies (Supplementary Fig. 1B). We immunized BALB/c mice intramuscularly with 2 μg of *mCherry*-LNPs, *BLP4*-LNPs, or *CDA1*-LNPs three times with two-week intervals (day -42, -28, and -14). Sera collected 2 weeks after each vaccination contained mCherry/Cda1-specific antibodies by western blot analysis and their level increased significantly after the first and second booster (Fig. 2A and Supplementary Fig. 2C). Thus, this prime-2 booster regimen yields high levels of antigen-specific antibodies.

To test if these mRNA-LNPs are protective against cryptococcosis, we challenged the immunized mice intranasally with a highly virulent clinical isolate H99 and monitored them for additional 40 days. Based on previous studies by us and others, H99-infected naïve mice would have succumbed to the infection within 4 weeks^15–17^. As expected, all *mCherry*-LNPs vaccinated mice reached clinical endpoints by DPI 26, with a median survival of 23 days (Fig. 2B). All *BLP4*-LNPs immunized mice succumbed to the infection by DPI 25. The *CDA1*-LNPs group showed slightly prolonged median survival (26 days), but the difference was not statistically significant. Since mouse background and intervals between shots could impact the vaccination effect, we tested mRNA-LNPs in CBA/J mice with 3 week intervals. As seen in BALB/c mice, all CBA/J mice immunized with *mCherry*-LNPs succumbed to infection by DPI 26 (Fig. 2C). The *CDA1*-LNPs and the *BLP4*-LNPs group had a median survival of 23 and 25 days. Again, *CDA1* and *BLP4* mRNA-LNPs by themselves at the tested dose offer no significant protection against cryptococcosis.

Cda1/Blp4 subunit protein vaccines become highly effective against cryptococcosis after encapsulation in glucan particles^8^. A recent study showed that a standard *RPL6*-LNP vaccine alone was ineffective against *Plasmodium berghei* sporozoites in mice, but the addition of an agonist resulted in effective protection^18^. We hypothesized that addition of an adjuvant might be necessary. As polysaccharide extracts have been used as adjuvants in experimental fungal vaccines^7^ and the cryptococcal capsule is predominantly made of glucuronoxylomannan and galactoxylomannan and is weakly immunogenic^19,20^, we tested the efficiency of our mRNA-LNP vaccines coupled with the purified capsule in CBA/J mice. The median survival for both the *mCherry*-LNPs+capsule group and the *BLP4*-LNPs+capsule group was 25 days, and the addition of the extracted capsule made no apparent difference (Fig. 2D). Remarkably, 80% of the mice immunized with *CDA1*-LNPs+capsule survived the entire study period. Moreover, the lung fungal burdens of the surviving *CDA1*-LNPs+capsule vaccinated mice were significantly lower at termination compared to the control mice which reached the clinical end points much earlier (Fig. 2E). These results demonstrate that *CDA1*-LNPs with the capsule adjuvant provide strong protection against lethal cryptococcal infection.

Antigens in mRNA vaccines may work differently from those in subunit protein vaccines. For example, for glycosylphosphatidylinositol (GPI)-anchored glycoproteins, only their middle part is typically expressed in *E. coli* for subunit protein vaccines. This is because both the N-terminal signal peptide and the hydrophobic C-terminus are cleaved, and the remaining C-terminus is anchored to membrane through the highly processed glycolipid GPI anchor in Golgi. The mRNA vaccine platform, however, allows the expression of the entire membrane anchored protein and likely post-translational modifications in the host, which resemble the native eukaryotic pathogen proteins. This idea is supported by the higher efficacy of GPI-anchored protein Pvs25 expressed in full length than the truncated versions as an mRNA vaccine against transmission of the malaria-causing parasite *Plasmodium vivax*^21^. In-host translation also eliminates the need and associated issues when purifying antigen proteins expressed in *E. coli* or tissue culture cells (e.g. toxic contaminants).

Adjuvants enhance immunogenicity of vaccines^22^. Glucan particles are yeast shells composed of primarily β-1,3-glucan, and they promote receptor-mediated uptake by antigen presenting cells and enhance immune responses to the encapsulated antigens^23^. Cda1 and Blp4 recombinant proteins encapsulated in glucan particles provide strong protection against cryptococcosis^8^. Here, we chose the cryptococcal capsule as the adjuvant. We found that *CDA1*-LNPs, but not *BLP4*-LNPs, when coupled with the extracted capsule, provided a remarkably strong protection against the otherwise lethal infection. It is possible that the capsule polysaccharides are augmenting the effect of the mRNA vaccine given that a variety of polysaccharide extracts are being used as adjuvants for fungal vaccines^7^. The idea of combining polysaccharides with mRNA vaccines has been recently proposed to be tested against bacterial infections^24,25^. That said, it is also possible that other residual molecules such as proteins in the extracted capsule could be the culprit. Whether it is the capsule polysaccharides, residual capsular proteins, or both that potentiated the protective effect of *CDA1*-LNPs is yet to be determined. Notably, we used 2 μg of mRNA in our vaccination experiments while most RNA-LNPs used in mice ranged from 2–100 μg^9^. Thus, we are hopeful that by selecting the strongest immunogens and appropriate adjuvants, and by optimizing the dose and vaccination intervals, mRNA-LNPs would be a promising platform for antifungal vaccines.

There are still many challenges ahead that need to be addressed in the future for using mRNA-LNP vaccine against any fungal disease^26^. For example, the populations at high risk of cryptococcosis are individuals with compromised immune systems, including HIV/AIDS patients, recipients of organ and stem cell transplants, long-term steroid therapy, and cancer patients. Thus, the ability to protect immunodeficient individuals is a must for successful cryptococcal vaccine development^1^. Although some whole cell vaccines can provide the protection in murine models of cryptococcosis with CD4^+^ T cell deficiency^17,27–29^, there has no report of such protection from subunit protein vaccines yet. Due to safety concerns (e.g. cross reactivity between fungal and host molecules), complex vaccines such as whole cell vaccines are unlikely to be adopted in the clinic despite their ability to elicit strong and lasting immunity^30^. Thus, testing the protective effect of mRNA-LNP vaccines with the strongest antigens and appropriate adjuvants in the immunocompromised animal model would be the next critical step. Genetic adjuvants encoding cytokines have been adopted in DNA-based vaccines and greatly enhance their efficacy^31,32^. Due to the intrinsic immune-stimulating characteristics and the co-delivery ability of the mRNA of immunostimulants in vivo, it is possible that employing a similar strategy in mRNA-LNP vaccines could be effective against fungal infections even in immunocompromised hosts.

## Methods

### Design and synthesis of mRNAs encoding candidate cryptococcal immunogens or the mCherry reporter control

Sequences encoding a mCherry fluorescent protein reporter control and two *C. neoformans* immunogens Cda1 and Blp4 were shown in Supplementary Table 1. The sequences of *C. neoformans* immunogens contain their endogenous signal peptides (SP) for secretion and ω sequences for a glycosylphosphatidylinositol (GPI) anchor, while the mCherry sequence has neither. The nucleotide codon compositions of the protein coding regions were optimized for efficient mRNA translation in mice and humans. To ensure efficient translation, we initially employed the 84 nt 5’ UTR from human cytoplasmic actin, *ACTB* (NM_001101.5) (Supplementary Table 1), and modified 5 bp to generate a more optimized Kozak sequence for improved translational efficiency. We used 3’ UTR from human hemoglobin alpha 1 based on its successful incorporation into Moderna’s Covid-19 spike protein mRNA-LNP vaccine design^9^. In our optimization process, we tested additional 5’ UTR sequences which, together with the corresponding primers were also included in Supplementary Table 1.

The DNA coding sequences (CDS) of the candidate immunogens and the mCherry reporter, and the flanking sequences were synthesized commercially (GeneScript) into the high-copy plasmid pUC57. The gene sequences were PCR amplified into a linear dsDNA template using a forward primer adding the T7 polymerase binding and initiation site, and a long reverse primer with a 120 bp poly(A) tail. Each immunogen encoding mRNA was transcribed from the T7 polymerase using APExBio’s HyperScribe® kit (Cat # K1064), during which the G_PPP_G cap was also added. During transcription, some of the normal uridine base nucleotides were replaced with N1-methyl-pseudouridine to make m1ψmRNA. The synthesized mRNA was purified by phenol-chloroform extraction and ethanol precipitation. Briefly, the reaction volume was adjusted by addition of nuclease-free water. 3 M sodium acetate was added, and the solution was mixed thoroughly. The volume ratio was 1:1 phenol/chloroform mixture extraction, followed by extraction twice with chloroform. The aqueous phase was collected and transferred to a new tube. RNA was precipitated with 2 volumes of ethanol and incubated at -20 °C for at least 30 min before being collected by centrifugation. The pellet was washed with cold 70% ethanol and resuspended in nuclease-free water. The concentration of RNA was measured by the RiboGreen reagent.

### Package N1ψmRNA in LNPs

SM102 lipid was included based on its use in Moderna’s Covid-19 Spike mRNA-LNP vaccine. The lipid combination includes the ionizable cationic amino-lipid SM-102 (ALC-0315, CAS 2089251-47-6, Broad Pharm, BP-25499), zwitterionic amino phospholipid DSPC (CAS 816-94-4, Broad Pharm, #BP-25623), a stealth amino phospholipid mPEG-2000-DSPE (CAS 1397695-86-1, Broad Pharm, Cat.# BP-25496), and cholesterol (CAS 57-88-0, Millipore Sigma Cat# PHR1533) in mol percent ratios of 50:10:1.5:38.5, respectively.

We employed microfluidic mixing to encapsulate mRNAs into LNPs, using PreciGenomes NanoGenerator’s medium scale Flex-M Nanoparticle Synthesis microfluidic device. 2 mg total lipids in ethanol and 100 μg mRNA in pH 4.0 buffer (25 mM acetate) were mixed together at a 3:1 volume ratio at 2 ml per min in PreciGenome’s microfluidic mixing chips^12^. Immediately after construction, the product mRNA lipid complexes were dialyzed into 100 volumes of pH 7.5 TAS2 buffer (20 mM Tris Base, 13 mM acetic acid, 8.7% w/v sucrose). Dialysis into neutral buffer is reported to convert mRNA lipid complexes into closed spherical LNPs. The efficiencies of RNA packaging and the sizes of generated liposomes varied slightly across different batches.

### Characterization of RNA-LNPs packaging

The efficiency of mRNA packaging in LNPs was quantified by comparing the RiboGreen fluorescence signal in lysed LNPs and unlysed LNPs. The LNPs were lysed in 0.5% Triton X100 in a microtiter plate. The RiboGreen reagent was used at the work concentration of 1:200 (Quant-iT™ RiboGreen™ RNA Reagent, Thermo Fisher, Waltham, MA, USA). The fluorescence of the RNAs bound by RiboGreen was measured using standard GFP filters (ex485/em528) and quantified by comparison to a standard curve made with RiboGreen binding to specified amount of purchased yeast RNAs. The efficiency of packaging (percentage) was calculated using Equation #1.

1 $$\% {Efficiency\; Packaging}=\frac{{FLU\; lysed\; sample}-{FLUunlysedLysed}}{{FLU\; lysed\; sample}}\,\times\,100$$

### Nanoparticle size characterization

The size distribution of LNPs was determined using two methods. The first method used a NanoFCM Flow Nanoanalyzer (NanoFCM INC., China). The quality control beads (250 nm Fluorescent Silica Microspheres) were pre-run to align and calibrate the NanoAnalyzer. NanoFCM Silica Nanospheres Cocktail #1 (S16M-Exo, diameter 68–155 nm) size standards (10^7^–10^12^ particles/ml) were pre-run as the size standards to create a calibration curve of the particle size and side scattering intensity. Then 100 μl diluted LNPs (10^6^–10^8^ particles/ml) were run to measure the concentration and size distribution of LNPs based on the calibration curve. The data were analyzed by program NF Profession 1.0. The second method is based on electron microscopy (EM). For negative-stain EM, 100 μl sample was pipetted onto a glow-discharged formvar/carbon-coated copper grid (Ted Pella) and stained with 1% pH-adjusted phosphotungstic acid (PTA) for 1 min. Negative-stain electron micrographs were acquired using a JEOL JEM1011 transmission electron microscope equipped with a high-contrast 2K-by-2K AMT midmount digital camera at the Georgia Electron Microscopy center. Liposome size measurements were made from EM images in Image J.

### Assessment of the ex vivo and in vivo activities of mCherry mRNA-LNPs

#### Cell transfection

Mouse macrophage cell line J774A.1 (ATCC TIB-67TM) was acquired from the American Type Culture Collection and cultured in Dulbecco’s modified Eagle’s medium (DMEM) (catalog no. 30-2002) with 10% fetal bovine serum (FBS). Freshly grown J774A.1 cells were seeded in 24-well microtiter plates (1 × 10^5^ cells per well) and cultured at 37 °C with 5% CO_2_ for 12 h. Then the macrophage culture medium was replaced with fresh DMEM medium containing 2.5 μg naked mRNA of mCherry or *mCherry*-LNPs for 24 h. Then the medium was removed, and cells were washed with DPBS. Washed cells were stained with 1 μg/ml Hoechst 33342 (62249, Thermo Fisher) for 20 min. The Echo Revolve R4 microscope (#RVSF1000) was used to visualize red fluorescent cells. Merged fluorescent images were taken using the DAPI channel (20% light intensity, 20 msec exposure) and the TRITC channel (100% light intensity, 285 msec exposure).

#### Animal injection

Female BALB/c mice and female CBA/J mice of 8–10 weeks old were purchased from the Jackson Laboratory (Bar Harbor, ME). Mice were sedated with ketamine and xylazine via intraperitoneal (i.p.) injection and then intermuscular (i.m.) administration of 50 μl *mCherry*-LNPs (2 μg of mRNAs) into the thighs of the mice. Mice were euthanized at 24 h after i.m. injection to examine the expression of the *mCherry*-LNPs. A 5 mm diameter area of muscle and subcutaneous fat surrounding the injection sites were excised. Approximately 1 mm thick sections of the tissue were manually made with a razor blade. Sections were stained with 1 μg/ml Hoechst 33342 (62249, Thermo Fisher) for 20 min and then pressed flat under a glass coverslip. The Echo Revolve R4 microscope (#RVSF1000) was used to visualize red fluorescent cells in the sections derived from the injected and mock injected mouse tissues. Merged fluorescent images were taken using the DAPI channel (20% light intensity, 20 msec exposure) and the TRITC channel (100% light intensity, 285 msec exposure).

### Purification of recombinant proteins

The plasmid harboring the mCherry or the Cda1 cDNA was transformed into *E. coli* strain BL21. The bacterial cells were first grown overnight at 37 °C in 5 ml LB medium containing kanamycin before being inoculated into 1 L LB containing kanamycin. When the OD_600_ reached 0.6 to 0.7, 1 mM isopropyl β-D-1-thiogalactopyranoside (IPTG) was added to induce the expression of mCherry/Cda1 at 37 °C for 4 h. The *E. coli* cells were collected, washed three times with PBS, and then lysed by French press. The lysate was centrifugated and the supernatant was used in affinity chromatography using Ni-NTA beads (Sigma). The purified protein was stored at -80 °C until further use.

### Western Blot

Purified mCherry or Cda1 proteins (2 μg) were mixed with 2X sampling buffer and boiled at 100 °C for 10 min. The protein samples were separated on 10% SDS-PAGE gels and then transferred to polyvinylidene difluoride membrane for incubation using serum (1:100) from *mCherry-*LNPs and *CDA1*-LNPs immunized mice at 4 °C overnight. The membrane was washed four times with PBST, and then incubated with the secondary antibody Goat Anti-Mouse IgG (H + L)-HRP Conjugate (BioRad, 1:10000) at 21 °C for 2 h. The blot was washed four times with PBST and then imaged by UVP ChemStudio (Analytic Jena).

### Capsule purification

The *C. neoformans* clinical isolate H99 was inoculated into 100 ml of YPD medium and incubated at 30 °C with agitation (150 rpm) for 5 days. Cells were then harvested, washed three times, and resuspended in 15 ml of dimethyl sulfoxide (DMSO). Cells were incubated at 21 °C with gentle agitation for 30 min and then centrifuged at 1000 x g for 5 min (Eppendorf centrifuge 5810 R). The supernatant was collected. An additional 15 ml of DMSO was added to the pellet for a second round of extraction, which was incubated again at 21 °C for 30 min. The cells were centrifuged once more at 1000 x g for 5 min, and the supernatant collected was combined with the supernatant from the first round. The samples were then thoroughly dialyzed against sterile water at 4 °C. The dialyzed samples were lyophilized, and the mass was determined in an analytical balance^33^. Then the samples were diluted in TAS2 buffer and stored at -20 °C.

### Vaccination against cryptococcosis in a murine model

Mice were vaccinated three times with 2 μg of *mCherry*-LNPs, *CDA1*-LNPs or *BLP4*-LNPs at day -42, day -28, and day -14 via i.m. injection. The numbers of the mice were indicated in the corresponding figure legends. For infection, live H99 cells with the initial inoculum of 10^6^ cells/ml were cultured in 3 ml of YPD medium at 30 °C with shaking 220 rpm for 15 h. Cells were washed with sterile saline three times and adjusted to a final density of 2 × 10^5^ cells/ml. Mice were sedated with ketamine and xylazine via intraperitoneal injection, and then inoculated intranasally with 50 μl fungal cells suspension (1 × 10^4^ cells/animal). After the challenge, mice were monitored daily for disease progression, including weight loss, labored breathing, gait changes and fur ruffing. For the survival experiments, mice were euthanized when they reached the defined clinical endpoints. All the surviving mice were terminated at the predetermined days for ending of the experiments unless all mice succumbed to the infection prior to the study termination. For serum collection, serum was sampled from mouse cheeks on the indicated days. The statistical significance of animal survival between groups was determined by the Mantel-Cox log-rank test.

## Supplementary information

1-10-25 Supplemental infornation

## Data Availability

Data is provided within the manuscript or supplementary information files. The DNA sequences are deposited in Genbank: PQ878553 and PQ878554.
