# Supplementary material for: Developing mRNA lipid nanoparticle vaccine effective for cryptococcosis in a murine model
Source: NPJ Vaccines. 2025 Feb 4;10:24. doi: 10.1038/s41541-025-01079-z (PMC11794474; doi:10.1038/s41541-025-01079-z)
Supplement: Supplementary file 1 — 1-10-25 Supplemental infornation [file 41541_2025_1079_MOESM1_ESM.pdf]

Developing mRNA lipid nanoparticle vaccine effective for cryptococcosis in a murine model

Yeqi Li[1], Suresh Ambati[2], Richard B. Meagher[2#], Xiaorong Lin[1#],

[1]Department of Microbiology, University of Georgia, Athens, GA. 30602.

[2]Department of Genetics, University of Georgia, Athens, GA. 30602.

[#]**Correspondence:** Richard Meagher meagher@uga.edu & Xiaorong Lin xiaorong.lin@uga.edu

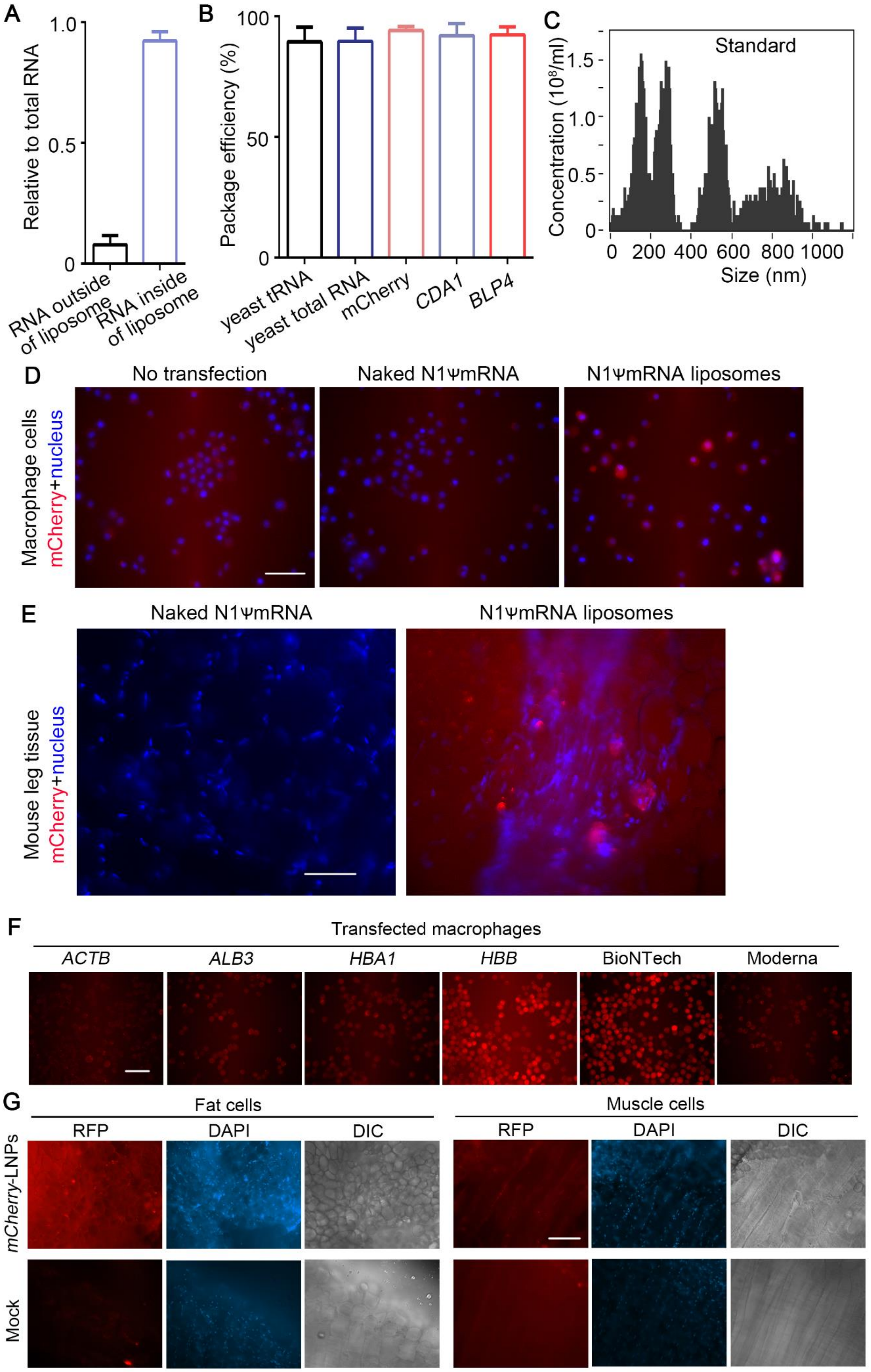

A
Relative to total RNA
1.0
0.5
0
RNA outside of liposome
RNA inside of liposome
B
Package efficiency (%)
100
50
0
yeast tRNA
yeast total RNA
mCherry
CDA1
BLP4
C
Standard
Concentration (10^8/ml)
1.5
1.0
0.5
0
0
200
400
600
800
1000
Size (nm)
D
No transfection
Naked N1ΨmRNA
N1ΨmRNA liposomes
Macrophage cells
mCherry+nucleus
E
Naked N1ΨmRNA
N1ΨmRNA liposomes
Mouse leg tissue
mCherry+nucleus
F
Transfected macrophages
ACTB
ALB3
HBA1
HBB
BioNTech
Moderna
G
Fat cells
Muscle cells
RFP
DAPI
DIC
RFP
DAPI
DIC
mCherry-LNPs
Mock

Fig S1. Validation of mRNA-LNPs using the mCherry reporter. (A-B) The RNA contents of complete LNPs and lysed LNPs were detected by the Ribogreen assay. (C) The analysis of the standard beads by Nanoanalyzer. (D) 2.5 µg of naked *mCherry* mRNA or *mCherry*-LNPs were transfected in murine macrophage J774A.1 cells and incubated for 24 h. Cells were stained with 1 µg/ml Hoechst 33342 for 20 min. Scale bar: 20 µm. (E) At 24 h post intramascular injection with mCherry-LNPs delivering 2 µg of mRNAs into the thighs of mice, muscle with associated subcutaneous fat tissue surrounding the injection sites were excised and manually sectioned. Cells were stained with 1 µg/ml Hoechst 33342 for 20 min. Scale bar: 90 µm. (F) The images of the mCherry signal in murine macrophage J774A.1 cells at 24 h after transfection with 2 µg *mCherry*-LNPs with different 5' UTRs. Scale bar: 20 µm. (G) After i.m. injection with 2 µg mCherry-LNPs made with the BioNTech 5' UTR construct into the thighs of mice for 24 h, muscle and subcutaneous fat tissues surrounding the injection sites were excised and manually sectioned. Cells were stained with 1 µg/ml Hoechst 33342 for 20 min. Scale bar: 90 µm.

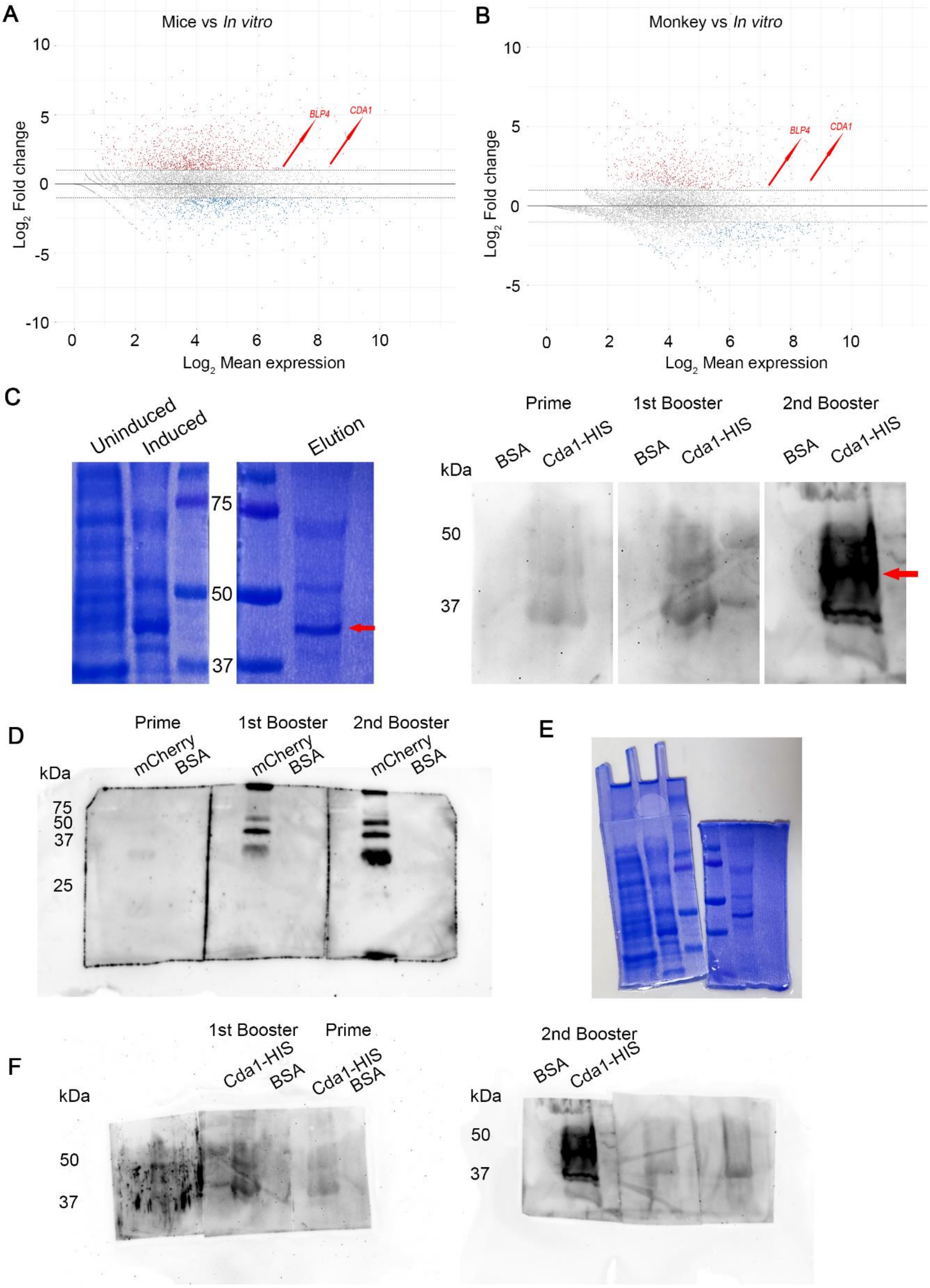

Fig S2. Optimizing 5'UTRs of *mCherry*-LNPs enhances the effective mCherry expression *ex vivo* and *in vivo.* (A-B) Volcano plot of differential gene expression of H99 between *in vitro* cultures in YPD medium and in mouse lungs (A), or in monkey lungs (B). (C) The Coomassie blue staining of the purified Cda1-His. Purified Cda1-HIS and the control

BSA proteins probed with serum from mice vaccinated with *CDA1*-LNPs. The prime and booster pictures are from the different membranes. The red arrow indicates the Cda1-HIS band. (D) The original picture for Figure 2A. (E) The original gel picture for Supplementary Figure 2C. (F) The original picture of membranes in Supplementary Figure 2C.

Supplementary Table 1 Optimal sequences and primers.

mCherry	ATGGTGAGCAAAGGAGAGGAGGACAACATGGCCATCATCAA<br>GGAGTTCATGAGGTTCAAAGTCCACATGGAGGGCTCGGTTA<br>ACGGTCATGAATTTGAGATTGAAGGGGAGGGTGAAGGGAGA<br>CCTTATGAGGGAACACAGACAGCCAAACTGAAAGTGACCAA<br>GGGCGGCCCCCTGCCCTTTGCCTGGGACATCCTTAGCCCTCA<br>GTTTATGTATGGCTCCAAGGCTTACGTTAAACACCCTGCTGA<br>CATCCCAGACTACCTGAAGTTGTCCTTCCCAGAAGGCTTCAA<br>GTGGGAAAGAGTGATGAACTTTGAAGATGGAGGAGTGGTGA<br>CTGTGACCCAAGACAGCTCCCTGCAGGACGGTGAATTCATC<br>TACAAGGTGAAGCTGCGGGGCACCAACTTCCCTAGTGACGG<br>CCCTGTCATGCAGAAGAAGACAATGGGCTGGGAGGCCAGCT<br>CTGAGAGGATGTACCCAGAGGATGGGGCCCTGAAGGGAGAA<br>ATCAAGCAGCGGCTGAAGCTAAAAGATGGCGGCCACTATGA<br>CGCAGAGGTAAAAACCACATACAAAGCCAAGAAACCAGTG<br>CAGCTGCCTGGGGCCTACAATGTCAACATCAAACTGGATATC<br>ACCTCTCACAATGAAGACTATACCATTGTGGAGCAGTACGAG<br>AGAGCTGAGGGCCGGCACAGCACAGGAGGCATGGATGAAC<br>TCTACAAG
CDA1	ATGTTCACTTTTGCCGCCTTCTCTGCCCTGCTCATCAGCTTGG<br>CCGGCGTGGTCGCTCAAACTACAGGCACCTCTGTGGACTCTT<br>CTATTTTGACCAAAACCGCCGACAGTACCGGGCCTTCCGGCT<br>TCAGTATCCCTGCCCTGTCAGAGCTGACCAGTGGCGCCCCCA<br>CAGACTCTACTGTGGCTCTCTACAGCACCTTCGCCGCCGGAG<br>CGACCCCTACCGTGTCCGGAGCCCCTGTGCTACCTACATCTG<br>CTCTGACTATCGCTGACTACCCAGCGCTGGACGTGACACCAC<br>CTACCAACAGTTCCCTGGTGAAGGACTGGATGGCCAAGATT<br>GACCTGTCCAAGGTGCCTAGCTACAACGTCACCACTGGGGA<br>CTGCAGCACAGACGCCGCAGCCATCTCTGACGGAAGATGCT<br>GGTGGACGTGTGGAGGCTGCACCCGCGAGACCGATATCGTA<br>GAGTGTCCAGATAAGAATGTGTGGGGCCTTAGCTATGATGAT<br>GGCCCTTCCCCCTTCACACCTCTGTTGATCGATTACCTGCAG<br>GAAAAGAACATCAAGACCACGTTCTTTGTAGTCGGCTCCCG<br>AGTACTGAGCAGACCTGAGATGCTGCAAACAGAGTACATGT<br>CAGGCCACCAGATTTCTATCCACACCTGGAGCCACCCAGCCC<br>TGACTACCCTGACAAATGAGGAGATCGTGGCAGAACTCGGC<br>TGGACCATGAAGGTTATCAAGGACACCCTGGGAGTGACCCC<br>CAACACCTTCAGGCCTCCCTACGGTGACATTGATGACAGAGT<br>GCGGGCTATTGCTGCCCAGATGGGCCTGACCCCAGTGATCTG<br>GACTAGTTACACTGATGGAAGTACCACGGTGAACTTTGACAC<br>CAATGACTGGCACATCAGCGGCGGCACAGCTACTGGCGCTT<br>CCTCTTATGAAACCTTTGAGAAAATCTTAACTGAATACGCTC<br>CTAAACTGGACACCGGATTCATTACTCTGGAGCATGACATCT<br>ACCAGCAGTCCGTGGATCTGGCTGTGGGCTACATCCTGCCCC

	AGGTGTTGGCCAACGGAACCTACCAACTGAAGAGCATCATC<br>AACTGCCTGGGCAAGGATACCAGCGAGGCCTACATTGAAAC<br>ATCCAGCAACCAGACAACGACCCAGATAACAGCTGCCACAG<br>GATCACAGAGCACATTCTTCCAGCCAATTGTAGGCACTGCCA<br>CCGGAGCCGAGGTGAGCGCCCCCTCAGAAGCCACTGGCTCT<br>ACAGCAGCGGGCAGCGCAGCGAGCACCACCAGCGGCTCTG<br>GGGCCAGCGCCTCGACCGGAGCCGCATCCAACACAAGCTCC<br>TCGGGCTCTGGCCGGAGCGCTACGATGGGAGGCGCCCTGAT<br>CGCTCTGGCCGCAGTGGCCGTGGGCATGGTGTATGTGGCA
BLP4	ATGTTTGCCAAGGCTGCTGTGATCGCACTGGCATCTGCCTCC<br>ATCGTTGCTGCCGCCCCTGTGAACTGTGCTAGAGCAAAGCC<br>AACCACTTATGATGAAGGCTACCTGGAGTCCTATGACTCTTA<br>CCATGCCAGGTACCTCGCCCTGTCCTGCTACAGCCAGCACAA<br>CACCACTTTCTTTGATGACTGCTGCCACCCTCTTCTGGCTAC<br>AGAGACCTTGGCAGACAACCGGGCCTCCTACTGCACACCCA<br>ACAGCACCGCCGTGGCCTCTGTGAATGCTACCATCGCTGAAG<br>CAACTGCTAGTGCCACAGCCTCTGCAGACATTGAGGCAGAA<br>AGTCAGTACAACAACGCTTCGAGCTATGCAGCCGAGGCTAC<br>CGCTCCTGTTACCACCTCTGCTGAGGCCACCGCCCCGGTGAC<br>AGCTAGCGCGGAAGCTACAGCCAGTGTGACCGCCGCTGCTG<br>TCAACAATGTGGCAGAGGTGGCCCAGCAGAGCGCCTCAGCC<br>AGCTCAGAGGAAGAGCAGCCAACTGCTAGCTCCTCTAGCAG<br>CTATGGAAGAGCCTCTGCCTCCTCTTCAAGCAGTGAGGAGG<br>AGTCGACATCAACTAGCAGCAGCTCTGCGAGTGATTCGTCG<br>AGCACCAGCAGCAGCCAGGTCTACACGGGGGGCTATGCCAC<br>CTTCTTCAGCCAAGGCGGTGTGGCTGGGGAGTGTGGAACTG<br>TCCACAGTGATGATGATTACGTCATCGCCATTGACAGCAATG<br>GCTGGTGGCAGGACTATGAATCTAATGACAGTTCGCCTTACT<br>GTGGCAAGCACATCACACTGACCAACACAAATAACGGCAAA<br>TCTGTGACCGCAGTAGTGGCAGATGTGTGCCCCACCTGTGA<br>GACGGCCAACTCTCTCGACCTGTCCATCGGAGCCTTCAACC<br>AAATCGCAACAGAAGAAGATGGCATGGTGCCCATCACCTGG<br>TACTTCACAGAC
ACTB<br>5’UTR	ACCGCCGAGACCGCGTCCGCCCCGCGAGCACAGAGCCTCGC<br>CTTTGCCGATCCGCCGCCCGTCCACACCCGCCGCGCCGCCAC<br>C
HBA1<br>3’UTR	GCTGGAGCCTCGGTGGCCTAGCTTCTTGCCCCTTGGGCCTCC<br>CCCCAGCCCCTCCTCCCCTTCCTGCACCCGTACCCCCGTGGT<br>CTTTGAATAAAGTCTGAGTGGGCGGCA
BioNTech<br>5’UTR	GCTGGAGCCTCGGTGGCCTAGCTTCTTGCCCCTTGGGCCTCC<br>CCCCAGCCCCTCCTCCCCTTCCTGCACCCGTACCCCCGTGGT<br>CTTTGAATAAAGTCTGAGTGGGCGGCA
HBB<br>5’UTR	ACATTTGCTTCTGACACAACTGTGTTCACTAGCAACCTCAAA<br>CAGCCACC

HBA1 5'UTR	ACTCTTCTGGTCCCCACAGACTCAGAGAGAACCCACC
ALB 5'UTR	CTAGCTTTTCTCTTCTGTCAACCCCACACGCCTTTGGCACA
Moderna 5'UTR	GGGAAATAAGAGAGAAAAGAAGAGTAAGAAGAAATATAAGACCCCGGCGCCGCCACC
T7 primer	CGAAATTAATACGACTCACTATAGGGGAATAAACTAGTATTCTT
PolyA primer	TTTTTTTTTTTTTTTTTTTTTTTTTTTTTTTTTTTTTTTTTTTTTTTTTTTTTTTTTTTTTTTTTTTTTTTTTTTTTTTTTTTTTTTTTTTTTTTTTTTTTTTTTTTTTTTTTTTTTTTTTTTTTTTTGCCGCCCACTCAGACTTTATTCAAA
Cda1-HIS-L	CCGCGCGGCAGCCATATGcagactacaggcacatcg
Cda1-HIS-R	GTGCTCGAGTGCGGCCGCctcaatgtatgcttcgga
